# *Ganoderma lucidum* ethanol extract inhibits the inflammatory response by suppressing the NF-κB and toll-like receptor pathways in lipopolysaccharide-stimulated BV2 microglial cells

**DOI:** 10.3892/etm.2013.895

**Published:** 2013-01-15

**Authors:** HYUN-MIN YOON, KYUNG-JUN JANG, MIN SEOK HAN, JIN-WOO JEONG, GI YOUNG KIM, JAI-HEON LEE, YUNG HYUN CHOI

**Affiliations:** 1 Departments of Acupuncture and Moxibustion, Dongeui University College of Oriental Medicine, Busan 614-052;; 2 Biochemistry, Dongeui University College of Oriental Medicine, Busan 614-052;; 3 Anti-Aging Research Center and Blue-Bio Industry RIC, Dongeui University, Busan 614-714;; 4 Faculty of Applied Marine Science, Cheju National University, Jeju 690-756;; 5 College of Natural Resources and Life Science, BK21 Center for Silver-Bio Industrialization, Dong-A University, Busan 604-714;; 6 Department of Biomaterial Control (BK21 program), Graduate School, Dongeui University, Busan 614-714, Republic of Korea

**Keywords:** *Ganoderma lucidum*, BV2 microglia, inflammation, nuclear factor κB, toll-like receptor

## Abstract

*Ganoderma lucidum* is a traditional Oriental medicine that has been widely used as a tonic to promote longevity and health in Korea and other Asian countries. Although a great deal of work has been carried out on the therapeutic potential of this mushroom, the pharmacological mechanisms of its anti-inflammatory actions remain unclear. In this study, we evaluated the inhibitory effects of *G. lucidum* ethanol extract (EGL) on the production of inflammatory mediators and cytokines in lipopolysaccharide (LPS)-stimulated murine BV2 microglia. We also investigated the effects of EGL on the LPS-induced activation of nuclear factor kappaB (NF-κB) and upregulation of toll-like receptor 4 (TLR4) and myeloid differentiation factor 88 (MyD88). Elevated levels of nitric oxide (NO), prostaglandin E_2_ (PGE_2_) and pro-inflammatory cytokine production were detected in BV2 microglia following LPS stimulation. We identifed that EGL significantly inhibits the excessive production of NO, PGE_2_ and pro-inflammatory cytokines, including interleukin (IL)-1β and tumor necrosis factor-α in a concentration-dependent manner without causing cytotoxicity. In addition, EGL suppressed NF-κB translocation and transcriptional activity by blocking IκB degradation and inhibiting TLR4 and MyD88 expression in LPS-stimulated BV2 cells. Our results indicate that the inhibitory effects of EGL on LPS-stimulated inflammatory responses in BV2 microglia are associated with the suppression of the NF-κB and TLR signaling pathways. Therefore, EGL may be useful in the treatment of neurodegenerative diseases by inhibiting inflammatory mediator responses in activated microglia.

## Introduction

A number of studies have indicated that mushrooms are rich sources of bioactive compounds. Among them, *Ganoderma lucidum* is a polypore mushroom that grows on the lower trunks of deciduous trees. This mushroom is a traditional Oriental medicine that has been widely used as a tonic to promote longevity and health for thousands of years in Asian countries, including China, Japan and Korea (1–4). The pharmacological activities of *G. lucidum*, particularly its intrinsic immunomodulating and antitumor properties, have been well-documented (4,5). Several studies have demonstrated that various *G. lucidum* extracts interfere with cell cycle progression, induce apoptosis and suppress angiogenesis in human cancer cells and thus act as anticancer agents (6–11). Additionally, it has also been suggested that *G. lucidum* extracts have a neuroprotective effect and may be useful in future studies on the pathogenesis and prevention of Alzheimer’s disease (AD) and Parkinson’s disease (PD) (12–15). However, the precise biochemical mechanisms underlying the *G. lucidum* extract-induced anti-inflammatory effects have not yet been clarified in microglial cells.

Microglia are the resident macrophage-like cells in the brain that play a major role in host defense and tissue repair in the central nervous system (CNS) (16,17). However, under pathological conditions, activated microglia release neurotoxic and pro-inflammatory mediators, including nitric oxide (NO), prostaglandin E_2_ (PGE_2_), reactive oxygen species and pro-inflammatory cytokines, including interleukin (IL)-1β, IL-6 and tumor necrosis factor (TNF)-α (18,19). Overproduction of these inflammatory mediators and cytokines causes severe neurodegenerative diseases, including AD, PD, cerebral ischemia, multiple sclerosis and trauma (20–22). Activated microglia are a major cellular source of pro-inflammatory and/or cytotoxic factors that cause neuronal damage in the CNS. Previous studies have also demonstrated that a decrease in the number of pro-inflammatory mediators in microglia may attenuate the severity of these disorders (23–25).

In the present study, we investigated the inhibitory effects of an ethanol extract of *G. lucidum* (EGL) and the mechanism of its anti-inflammatory action against the lipopolysaccharide (LPS)-stimulated pro-inflammatory responses in murine BV2 microglia. Our results indicate that EGL inhibits inflammatory reactions by inhibiting nuclear factor κB (NF-κB) and toll-like receptor (TLR) signaling pathways, suggesting that EGL may be useful for the treatment of neuroinflammatory and neurodegenerative diseases.

## Materials and methods

### Preparation of EGL

EGL was supplied by Dongeui University Oriental Hospital (Busan, Korea). The freeze-dried and milled *G. lucidum* fruiting bodies (200 g) were extracted with 25% ethanol (4 liters) at room temperature for 10 h using a blender. The extracts were filtered through a Whatman no. 2 filter (Maidstone, UK), concentrated to 500 ml under vacuum conditions and then stored at −20°C (11). The EGL solution was directly diluted in medium prior to assay.

### Cell culture

The murine BV2 cell line was maintained in Dulbecco’s modified Eagle’s medium supplemented with 10% fetal bovine serum, 100 U/ml penicillin and 100 *μ*g/ml streptomycin (Gibco-BRL, Grand Island, NY, USA) at 37°C in a humidified incubator with 5% CO_2_. The cells were pre-treated with the indicated EGL concentrations for 1 h before adding LPS (0.5 *μ*g/ml, Sigma-Aldrich, St. Louis, MO, USA). Cell viability was evaluated by the 3-(4,5-dimethylthiazol-2-yl)-2,5-diphenyltetrazolium bromide (MTT; Sigma-Aldrich) reduction assay. In brief, cells (1×10^5^ cells/ml) were seeded and treated with EGL and/or LPS for the indicated time periods. Following treatment, the medium was removed and the cells were incubated with 0.5 mg/ml MTT solution. After a 3 h incubation at 37°C in 5% CO_2_, the supernatant was removed and formation of formazan was measured at 540 nm with a microplate reader (Dynatech MR-7000; Dynatech Laboratories Inc., El Paso, TX, USA).

### NO production

NO concentrations in culture supernatants were determined by measuring nitrite, which is a major stable product of NO, using Griess reagent (Sigma-Aldrich). Cells (5×10^5^ cells/ml) were stimulated in 24-well plates for 24 h, then 100 *μ*l culture medium was mixed with an equal volume of Griess reagent (1% sulfanilamide, 0.1% N-(1-naphthyl)-ethylenediamine dihydrochloride and 2.5% H_3_PO_4_). Nitrite levels were determined using an enzyme-linked immunosorbent assay (ELISA) plate reader at 540 nm and nitrite concentrations were calculated by reference to a standard curve generated using known concentrations of sodium nitrite (26).

### Measurement of PGE_2_ production

BV2 cells were sub-cultured in 6-well plates (5×10^5^ cells/ml) and incubated with the indicated concentrations of EGL in the presence or absence of LPS (0.5 *μ*/ml) for 24 h. A 100 *μ*l aliquot of the culture-medium supernatant was collected for determination of the PGE_2_ concentration by ELISA (Cayman Chemical, Ann Arbor, MI, USA).

### RNA isolation and reverse transcription-polymerase chain reaction (RT-PCR)

Total RNA was isolated from the cells using the TRIzol reagent (Invitrogen Life Technologies, Carlsbad, CA, USA). The total RNA (1.0 *μ*g) was reverse-transcribed using M-MLV reverse transcriptase (Promega Corporation, Madison, WI, USA) to produce cDNAs. The inducible nitric oxide synthase (iNOS), cyclooxygenase (COX)-2, IL-1β and TNF-α genes were amplified from the cDNA using PCR. The PCR primers were as follows: mouse iNOS, 5′-ATG TCC GAA GCA AAC ATC AC-3′ and 5′-TAA TGT CCA GGA AGT AGG TG-3′; COX-2, 5′-CAG CAA ATC CTT GCT GTT CC-3′ and 5′-TGG GCA AAG AAT GCA AAC ATC-3′; IL-1β, 5′-ATG GCA ACT GTT CCT GAA CTC AAC T-3′ and 5′-TTT CCT TTC TTA GAT ATG GAC AGG AC-3′; and TNF-α, 5′-ATG AGC ACA GAA AGC ATG ATC-3′ and 5′-TAC AGG CTT GTC ACT CGA ATT-3′. Following amplification, the PCR products were electrophoresed on 1% agarose gels and visualized by ethidium bromide (Sigma-Aldrich) staining. Glyceraldehyde-3-phosphate dehydrogenase was used as an internal control.

### Protein extraction and western blot analysis

Cells were washed three times with phosphate-buffered saline (PBS) and lysed in lysis buffer (1% Triton X-100, 1% deoxycholate and 0.1% NaN3) containing protease inhibitors (Roche Diagnostics, Mannheim, Germany). In a parallel experiment, nuclear and cytosol proteins were prepared using NE-PER^®^ nuclear and cytoplasmic extraction reagents (Pierce Biotechnology Inc., Rockford, IL, USA) according to the manufacturer’s instructions. For the western blot analysis, equal amounts of protein were subjected to electrophoresis on sodium dodecyl sulfate-polyacrylamide gels and transferred to nitrocellulose membranes (Schleicher & Schuell Inc., Keene, NH, USA) by electroblotting. Blots were probed with the desired antibodies for 1 h, incubated with the diluted enzyme-linked secondary antibodies and visualized by enhanced chemiluminescence (Amersham Co., Arlington Heights, IL, USA) according to the manufacturer’s instructions. Actin and nucleolin were used as internal controls for the cytosolic and nuclear fractions, respectively. Antibodies against iNOS, COX-2, IκB-α, NF-κB p65, TLR4 and myeloid differentiation factor 88 (MyD88) were purchased from Santa Cruz Biotechnology (Santa Cruz, CA, USA). Antibodies against actin and nucleolin were obtained from Sigma-Aldrich. Peroxidase-labeled goat anti-rabbit immunoglobulin and fluorescein isothiocyanate (FITC)-conjugated donkey anti-rabbit IgG were purchased from Amersham Co. and Sigma-Aldrich, respectively.

### Cytokine assays

The levels of IL-1β and TNF-α were measured using ELISA kits (R&D Systems, Minneapolis, MN, USA) according to the manufacturer’s instructions. Briefly, BV2 cells (5×10^5^ cells/ml) were plated in 24-well plates and pre-treated with the indicated EGL concentrations for 1 h prior to treatment with 0.5 *μ*g/ml LPS for 24 h. A 100 *μ*l aliquot of each culture-medium supernatant was collected for determination of the IL-1β and TNF-α concentrations by ELISA.

### NF-κB luciferase assay

A total of 1×10^6^ BV2 cells were transfected with 2 *μ*g NF-κB-luciferase reporter plasmids (BD Biosciences, San Jose, CA, USA) using Lipofectamine according to the manufacturer’s instructions (Gibco-BRL). Then, the cells were pre-incubated in the presence or absence of EGL before being stimulated with LPS for 6 h. Cells were washed twice with PBS and lysed with reporter lysis buffer (Promega Corporation). Following vortexing and centrifuging at 12,000 × g for 1 min at 4°C, the supernatant was stored at −70°C. For the luciferase assay, 20 *μ*l cell extract was mixed with 100 *μ*l luciferase assay reagent at room temperature, then, the mixture was analyzed using a LB96V microplate luminometer (Perkin-Elmer, Waltham, MA, USA) (27).

### Statistical analysis

Data are presented as mean ± standard deviation. Statistical significance was determined using an analysis of variance followed by the Student’s t-test. P<0.05 was considered to indicate a statistically significant difference.

## Results

### Effects of EGL on NO and PGE_2_ production in LPS-stimulated BV2 microglia

**Figure 1.**
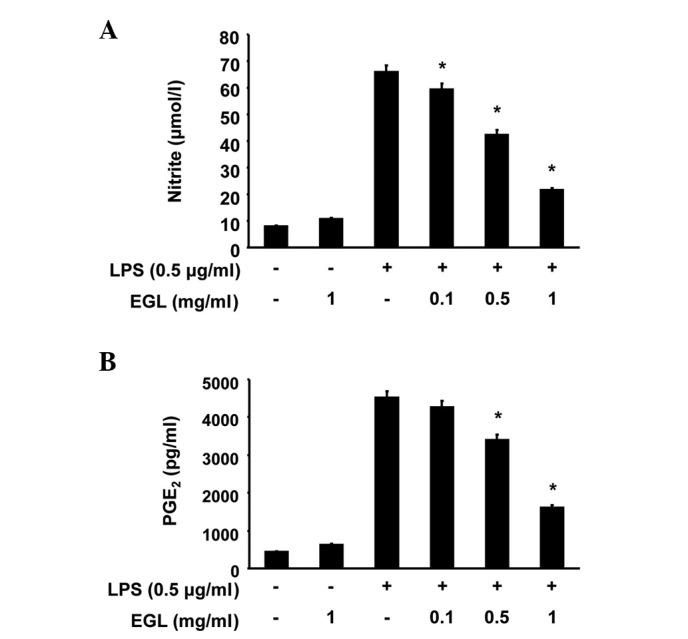
Inhibition of NO and PGE_2_ production by EGL in LPS-stimulated BV2 microglia. BV2 cells were pre-treated with various concentrations of EGL (0.1, 0.5 and 1.0 mg/ml) for 1 h before incubation with LPS (0.5 *μ*g/ml) for 24 h. (A) Nitrite content was measured using the Griess reaction and (B) PGE_2_ concentration was measured in culture media using a commercial ELISA kit. Each value indicates the mean ± standard deviation (SD) of three independent experiments. ^*^P<0.05 vs. cells treated with LPS in the absence of EGL. NO, nitric oxide; PGE_2_, prostaglandin E_2_; EGL, ethanol extract of *Ganoderma lucidum*; LPS, lipopolysaccharide; ELISA, enzyme-linked immunosorbent assay.

The potential anti-inflammatory properties of EGL were evaluated against the production of two major inflammatory mediators, NO and PGE_2_, in LPS-stimulated BV2 microglia. To determine the levels of NO and PGE_2_ production, the amounts of nitrite and PGE_2_ released into the culture medium were measured using Griess reagent and ELISA, respectively. According to the NO detection assay result, LPS alone markedly induced NO production from cells compared with the control (Fig. 1A). However, pre-treatment with EGL significantly repressed the levels of NO production in the LPS-stimulated BV2 microglia in a concentration-dependent manner, up to 1 *μ*g/ml. Under the same conditions, stimulating the cells with LPS also resulted in a significant increase in PGE_2_ production; however, this was markedly repressed by pre-treatment with EGL (Fig. 1B).

### Effects of EGL on cell viability in LPS-stimulated BV2 microglia

**Figure 2.**
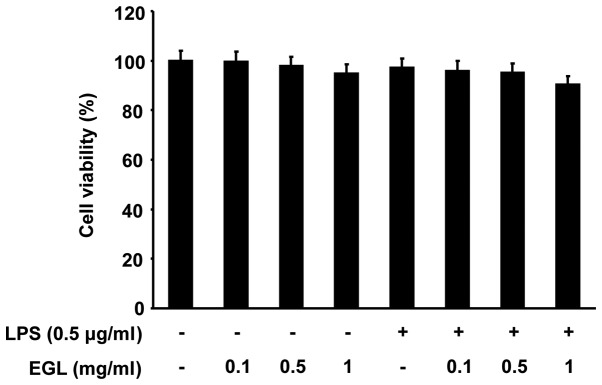
Effects of EGL and LPS on the cell viability of BV2 microglia. Cells were treated with the indicated concentrations of EGL or pre-treated with EGL for 1 h prior to LPS (0.5 *μ*g/ml) treatment, After 24 h, cell viability was assessed using an MTT reduction assay. The results are expressed as the percentage of surviving cells vs. control cells (no addition of EGL and LPS). Each value is the mean ± SD of three independent experiments. EGL, ethanol extracts of *Ganoderma lucidum*; LPS, lipopolysaccharide; MTT, 3-(4,5-dimethylthiazol-2-yl)-2,5-diphenyltetrazolium bromide; SD, standard deviation.

The cells were exposed to EGL for 24 h in the presence or absence of LPS to exclude a cytotoxic effect of EGL on BV2 cell growth. Cell viability was then measured by the MTT assay. As indicated in Fig. 2, the concentrations of EGL (0.1–1 mg/ml) used to inhibit NO and PGE_2_ production did not affect cell viability. The results clearly indicate that the inhibition of NO and PGE_2_ production in the LPS-stimulated BV2 cells was not due to a cytotoxic effect of EGL.

### Effects of EGL on LPS-stimulated iNOS and COX-2 expression

**Figure 3.**
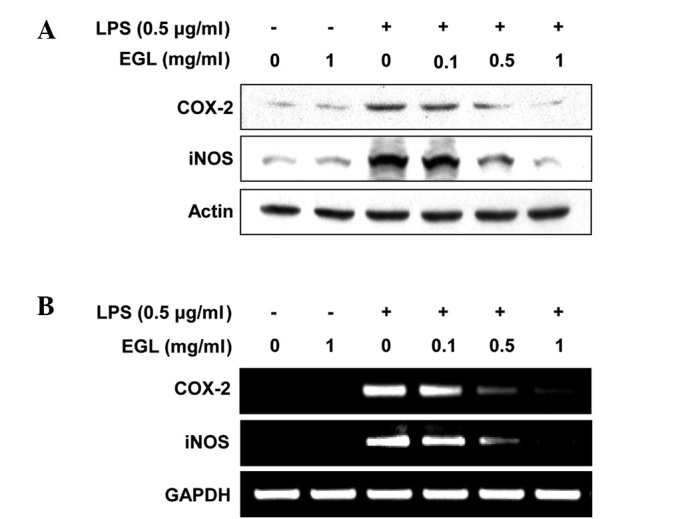
Inhibition of iNOS and COX-2 expression by EGL in LPS-stimulated BV2 microglia. (A) BV2 microglia were pre-treated with the indicated concentrations of EGL 1 h prior to incubation with LPS (0.5 *μ*g/ml) for 24 h. Cell lysates were then prepared and western blotting was performed using antibodies specific for murine iNOS and COX-2. (B) After LPS treatment for 6 h, total RNA was prepared for RT-PCR analysis of iNOS and COX-2 gene expression. Actin and GAPDH were used as internal controls for the western blot analysis and RT-PCR assays, respectively. iNOS, inducible nitric oxide synthase; COX-2, cyclooxygenase-2; EGL, ethanol extract of *Ganoderma lucidum*; LPS, lipopolysaccharide; RT-PCR, reverse transcription-polymerase chain reaction; GAPDH, glyceraldehyde 3-phosphate dehydrogenase.

RT-PCR and western blot analyses were carried out to determine whether the inhibition of NO and PGE_2_ production by EGL in LPS-stimulated BV2 cells was associated with reduced levels of iNOS and COX-2 expression. As shown in Fig. 3, iNOS and COX-2 protein levels were markedly upregulated following 24 h of treatment with LPS (0.5 *μ*g/ml) alone; however, EGL significantly inhibited the iNOS and COX-2 protein expression in the LPS-stimulated BV2 microglia in a concentration-dependent manner (Fig. 3A). The effects of EGL on iNOS and COX-2 mRNA expression were evaluated to investigate whether EGL suppressed the LPS-mediated induction of iNOS and COX-2 at the pre-translational level. RT-PCR data revealed that the reduction in iNOS and COX-2 mRNAs correlated with the reduction in the corresponding protein levels (Fig. 3B). These results suggest that EGL-induced reductions in iNOS and COX-2 expression were the cause of the reduced NO and PGE_2_ production levels.

### Effects of EGL on LPS-induced IL-1β and TNF-α production and mRNA expression

**Figure 4.**
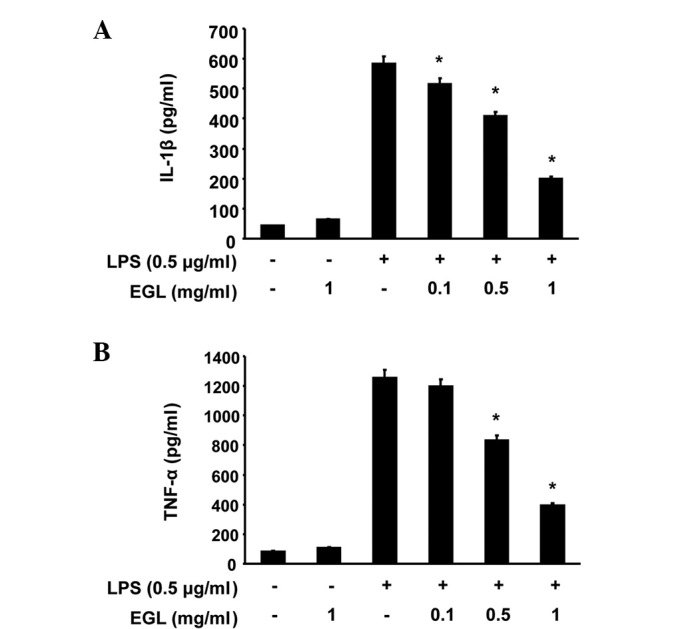
Effects of EGL on LPS-stimulated IL-1β and TNF-α production in BV2 microglia. BV2 cells were pre-treated with various concentrations of EGL for 1 h prior to LPS treatment (0.5 *μ*g/ml). After 24 h incubation, the levels of (A) IL-1β and (B) TNF-α present in the supernatants were measured. Each value indicates the mean ± SD of three independent experiments. ^*^P<0.05 vs. cells treated with LPS in the absence of EGL. EGL, ethanol extract of *Ganoderma lucidum*; LPS, lipopolysaccharide; IL, interleukin; TNF, tumor necrosis factor; SD, standard deviation.

**Figure 5.**
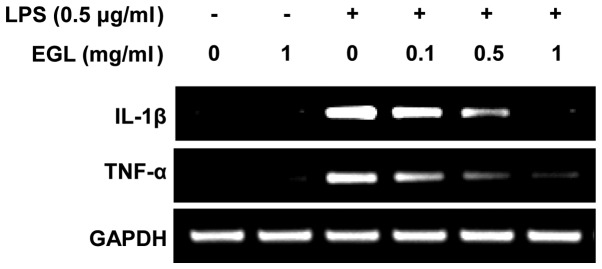
Effects of EGL on LPS-stimulated IL-1β and TNF-α expression in BV2 microglia. BV2 cells were pre-treated with the indicated concentrations of EGL for 1 h prior to LPS treatment (0.5 *μ*g/ml) and total RNA was isolated 6 h after LPS treatment. The levels of IL-1β and TNF-α mRNA were determined using RT-PCR. GAPDH was used as an internal control. EGL, ethanol extract of *Ganoderma lucidum*; LPS, lipopolysaccharide; IL, interleukin; TNF, tumor necrosis factor; RT-PCR, reverse transcription-polymerase chain reaction; GAPDH, glyceraldehyde 3-phosphate dehydrogenase.

The effects of EGL on the production of pro-inflammatory cytokines, including IL-1β and TNF-α, were analyzed using ELISA. BV2 cells were incubated with various concentrations of EGL in the presence or absence of LPS (0.5 *μ*g/ml) for 24 h and cytokine levels in the culture media were measured. As shown in Fig. 4, the IL-1β and TNF-α levels were markedly increased in the culture media of the LPS-stimulated BV2 microglia. However, pre-treatment with EGL significantly reduced the release of these pro-inflammatory cytokines in a concentration-dependent manner. In a parallel experiment, the effects of EGL on LPS-induced IL-1β and TNF-α mRNA expression were studied using RT-PCR. As shown in Fig. 5, IL-1β and TNF-α mRNA transcription levels also decreased following EGL treatment. These results suggest that EGL was effective in suppressing pro-inflammatory cytokine production by altering IL-1β and TNF-α transcription levels in activated microglia.

### Effects of EGL on LPS-induced NF-κB activity

**Figure 6.**
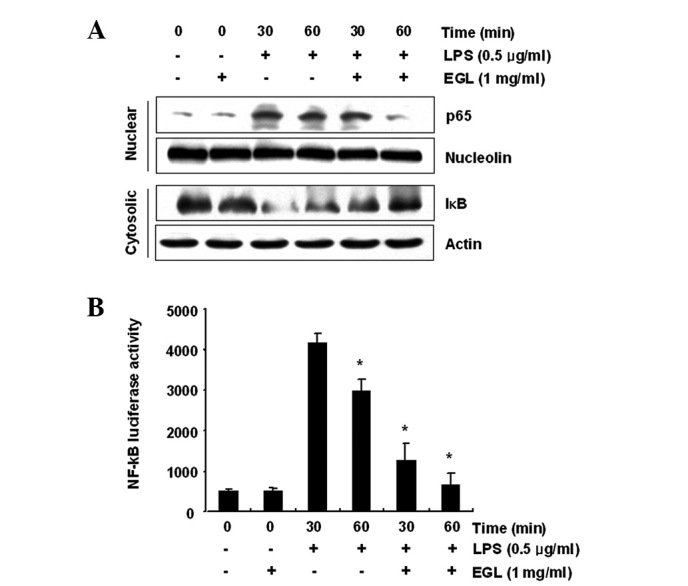
Effects of EGL on LPS-induced NF-κB translocation, IκB degradation and NF-κB activation in BV2 microglia. (A) Cells were treated with EGL (1 mg/ml) for 1 h prior to LPS treatment (0.5 *μ*g/ml) for the indicated times. Nuclear and cytosolic proteins were subjected to 10% SDS-polyacrylamide gel electrophoresis followed by western blotting using anti-NF-κB p65 and anti-IκB-α antibodies. Nucleolin and actin were used as internal controls for the nuclear and cytosolic fractions, respectively. (B) Transfected BV2 microglia with NF-κB-luciferase reporter plasmids were pre-treated with EGL (1.0 mg/ml) for 0.5 or 1 h and then stimulated with LPS (0.5 *μ*g/ml) for 0.5 or 1 h. NF-κB activity was expressed as luciferase activity. Each value is the mean ± SD of three independent experiments. ^*^P<0.05 vs. cells treated with LPS in the absence of EGL. EGL, ethanol extract of *Ganoderma lucidum*; LPS, lipopolysaccharide; NF, nuclear factor; SDS, sodium dodecyl sulfate; SD, standard deviation.

Activation of NF-κB is crucial for the induction of iNOS, COX-2, TNF-α and IL-1β genes in activated BV2 microglia (28,29). To further characterize the mechanism through which EGL inhibits pro-inflammatory and/or cytotoxic factor expression, the ability of EGL to prevent translocation of the NF-κB p65 subunit to the nucleus was first examined. Western blot analysis revealed that the amount of NF-κB p65 in the nucleus markedly increased following exposure to LPS alone; however, the LPS-induced p65 level in the nuclear fraction was reduced following EGL pre-treatment (Fig. 6A). In addition, the ability of EGL to block the LPS-stimulated degradation of IκB-α was investigated by western blotting. As shown in Fig. 6A, IκB-α was markedly degraded at 30 min after LPS treatment; however, this LPS-induced IκB-α degradation was significantly reversed by EGL. Furthermore, the inhibition of LPS-induced NF-κB activation by EGL was confirmed using a luciferase assay. BV2 cells transfected with NF-κB-luciferase reporter plasmids were pre-treated with EGL for 1 h then, following stimulation with LPS for 6 h, luciferase activity was measured. As shown in Fig. 6B, LPS significantly enhanced the NF-κB activity up to ∼8-fold over the basal level, whereas EGL significantly inhibited the LPS-induced NF-κB activity. These findings show that the anti-inflammatory effect of EGL in LPS-stimulated BV2 cells involves the NF-κB pathway.

### Effects of EGL on the levels of LPS-induced TLR4 and MyD88

**Figure 7.**
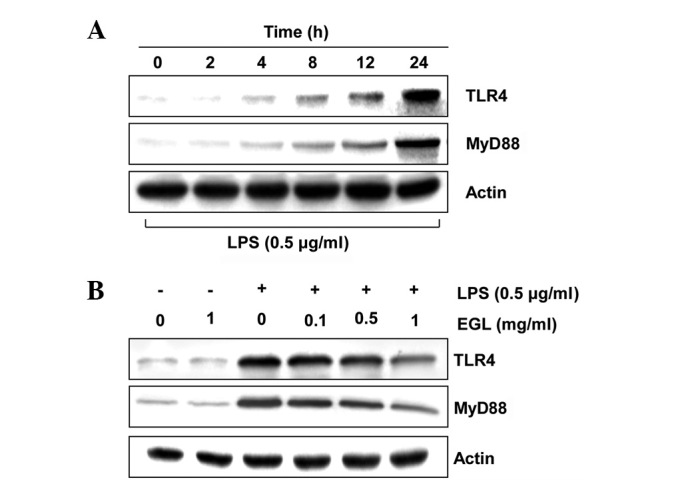
Effects of EGL on LPS-induced TLR4 and MyD88 expression in BV2 microglia. (A) Cells were treated with 0.5 *μ*g/ml LPS for the indicated times. The cells were sampled, lysed and 50 *μ*g proteins were separated by 10% SDS-polyacrylamide gel electrophoresis. (B) BV2 cells were pre-treated with 1.0 mg/ml EGL for 1 h prior to LPS treatment (0.5 *μ*g/ml) and total proteins were isolated 24 h after LPS treatment. The proteins were subjected to 10% SDS-polyacrylamide gel electrophoresis followed by western blotting using anti-TLR4 and anti-MyD88 antibodies and an ECL detection system. Actin was used as an internal control. EGL, ethanol extract of *Ganoderma lucidum*; LPS, lipopolysaccharide; TLR, toll-like receptor; SDS, sodium dodecyl sulfate; ECL, enhanced chemiluminescence; MyD88, myeloid differentiation factor 88.

Previous studies established that the inflammatory response to LPS completely relies on the presence of TLR4, which triggers the intracellular association of MyD88 with its cytosolic domain (30–32). To investigate whether the anti-inflammatory activity of EGL is associated with the modulation of these proteins, the effects of EGL on LPS-induced upregulation of TLR4 and MyD88 in BV2 cells were examined. As shown in Fig. 7, EGL concentration-dependently attenuated the LPS-stimulated increase in TLR4 and MyD88 expression. This finding indicates that EGL is capable of disrupting key signal transduction pathways, including TLR signaling pathways activated by LPS in BV2 microglia, which subsequently prevents the production of pro-inflammatory mediators and cytokines.

## Discussion

*G. lucidum* is an edible basidiomycete white-rot macrofungus mushroom that has long been prescribed to prevent and treat various human diseases, particularly in Asian countries (1–4). Extensive study has been carried out on the therapeutic potential of *G. lucidum*. The exact ingredients in *G. lucidum* have not yet been identified; however, the major active ingredients include polysaccharides, triterpenoids, unsaturated fatty acids and ergosterol (2,33–35). Although previous reports have indicated that *G. lucidum* extracts exhibit inhibitory actions on neurotoxicity (12–15), the anti-inflammatory and related immune responses remain poorly understood. Therefore, we investigated the inhibitory effects of EGL on the production of LPS-stimulated pro-inflammatory mediators and cytokines in BV2 microglia to evaluate the cellular and molecular mechanisms of the anti-inflammatory effects of this traditional medicine.

COXs are enzymes that catalyze the conversion of arachidonic acid to PGH_2_, which is the precursor of a variety of biologically active mediators, including PGE_2_, prostacyclin and thromboxane A_2_. COXs exist as two major isozymes: COX-1, a constitutive COX, and COX-2, an isoform that is induced during the responses to a number of stimulants and is activated at the inflammation site (36,37). Several studies have reported that COX-2 is associated with cytotoxicity in brain diseases, since the inhibition of COX-2 induction and/or activity reduces brain injury following ischemia and slows the progression of AD and PD (38). Additionally, NO is an important regulatory molecule in diverse physiological functions, including vasodilation, neural communication and host defense (39,40). In mammalian cells, NO is synthesized from three different isoforms of NOS, including endothelial NOS, neuronal NOS and iNOS. Activated microglial cells are a major cellular source of iNOS in the brain and the excessive release of NO by activated microglia is correlated with the progression of neurodegenerative disorders. The major producers of TNF-α in the brain are microglia and they may play a role in a number of pathological conditions of the brain (17,41,42). Therefore, TNF-α overexpression has been implicated in the pathogenesis of several human CNS disorders (18,43,44). IL-1β is also a potent pro-inflammatory cytokine that acts through IL-1 receptors on numerous cell types, including neurons and microglia. Moreover, IL-1β is an important mediator of neuroimmune interactions that participate directly in neurodegeneration (45). Thus, inhibiting inflammatory mediator and cytokine production or function serves as a key mechanism for identifying a treatment for inflammatory diseases, including brain injury. In the present study, EGL significantly suppressed LPS-stimulated PGE_2_ and NO production in BV2 microglia in a concentration-dependent manner without affecting cell viability (Figs. 1 and 2), which appeared to be due to the transcriptional suppression of COX-2 and iNOS (Fig. 3). Our data also indicate that treatment with EGL prior to LPS significantly attenuates the production of the inflammatory cytokines TNF-α and IL-1β, by inhibiting their expression at the transcriptional level (Figs. 4 and 5). These results suggest that the anti-inflammatory activity of EGL may contribute to the suppressive effects of COX-2, iNOS, TNF-α and IL-1β in LPS-activated BV2 microglia.

NF-κB, as a result of its key role in several pathological conditions, is a major drug target for a variety of diseases. NF-κB is also a primary regulator of genes that are involved in the production of pro-inflammatory cytokines and enzymes involved in the process of inflammation (28,29). NF-κB is normally located in the cytoplasm where it is complexed with an inhibitory IκB protein. In response to pro-inflammatory stimuli, IκB is phosphorylated and subsequently degraded. NF-κB is then released and translocated to the nucleus where it promotes the expression of inflammation-related genes. Moreover, blocking the transcriptional activity of NF-κB in microglial nuclei also suppresses the expression of iNOS, COX-2 and pro-inflammatory cytokines, including IL-1β and TNF-α (46,47). Our results indicate that EGL inhibits LPS-induced IκB-α degradation, nuclear translocation of the NF-κB p65 subunit and NF-κB transcriptional activity in BV2 microglia (Fig. 6). Therefore, inhibition of the NF-κB signaling pathway in microglia by EGL may result in the downregulation of pro-inflammatory mediators, resulting in an anti-inflammatory effect.

TLR family members are receptors of the innate immune system that recognize pathogen-associated molecular patterns. Among them, TLR4 acts as a major LPS signaling receptor, leading to the activation of key transcription factors, including NF-κB and activator protein (AP)-1, which, in turn, enhance the synthesis of effector inflammatory genes, including cytokines and chemokines (30–32,48). Stimulation of the TLR4 extracellular domain by LPS or endotoxins sequentially triggers the intracellular association of MyD88 with its cytosolic domain (30,49). Therefore, MyD88 serves as a key TLR4 adaptor protein, linking the receptors to downstream kinases, suggesting that TLR4 and MyD88 act as specific targets for inflammatory responses (30,48,50). Our data clearly demonstrate that EGL markedly inhibits LPS-induced TLR4 and MyD88 expression in BV2 microglia (Fig. 7). These results suggest that TLR4 and MyD88 are involved in the inhibitory effects of EGL on the LPS-induced expression of NO, PGE_2_ and pro-inflammatory cytokines.

In summary, we identified that EGL significantly attenuates the LPS-induced release of inflammatory mediators and cytokines, including NO, PGE_2_, TNF-α and IL-1β. Moreover, it acts at the transcriptional level in BV2 microglia. The anti-inflammatory action of EGL is mediated by the prevention of the activation of NF-κB and inhibition of IκB-degradation and possibly by inhibition of the TLR signaling pathway. Thus, EGL may provide an effective treatment for a number of neurodegenerative diseases; however, the pharmacology and mode of action of its active components require further investigation.
